# Frequency of the *CYP*2C19*17 polymorphism in a Chilean population and its effect on voriconazole plasma concentration in immunocompromised children

**DOI:** 10.1038/s41598-019-45345-2

**Published:** 2019-06-20

**Authors:** N. Espinoza, J. Galdames, D. Navea, M. J. Farfán, C. Salas

**Affiliations:** 1 Laboratorio Clínico, Hospital Dr. Luis Calvo Mackenna, Santiago, Chile; 2 Departamento de Pediatría y Cirugía Infantil, Campus Oriente, Hospital Dr. Luis Calvo Mackenna, Facultad de Medicina, Universidad de Chile, Santiago, Chile

**Keywords:** Paediatric cancer, Paediatric research

## Abstract

Invasive fungal infections (IFIs) are the most frequent cause of morbidity and mortality in immunocompromised children. Voriconazole is the first-line antifungal choice in the treatment of IFIs like aspergillosis. Voriconazole pharmacokinetics vary widely among patients and voriconazole is metabolized mainly in the liver by the CYP2C19 enzyme, which is highly polymorphic. The *CYP*2C19*17 allele is characterized by the presence of four single nucleotide polymorphisms expressing an ultra-rapid enzyme phenotype with an accelerated voriconazole metabolism, is associated with low (sub-therapeutic) plasma levels in patients treated with the standard dose. Considering that in our center a high percentage of children have sub-therapeutic levels of voriconazole when treated with standard doses, we sought to determine the frequency of the CYP2C19*17 polymorphism (rs12248560) in a Chilean population and determine the association between voriconazole concentrations and the rs12248560 variant in immunocompromised children. First, we evaluated the frequency of the rs12248560 variant in a group of 232 healthy Chilean children, and we found that 180 children (77.6%) were non-carriers of the rs12248560 variant, 49 children (21.1%) were heterozygous carriers for rs12248560 variant and only 3 children (1.3%) were homozygous carriers for rs12248560 variant, obtaining an allelic frequency of 12% for variant in a Chilean population. To determine the association between voriconazole concentrations and the rs12248560 variant, we analyzed voriconazole plasma concentrations in a second group of 33 children treated with voriconazole. In these patients, carriers of the rs12248560 variant presented significantly lower voriconazole plasma concentrations than non-carriers (*p* = 0,011). In this study, we show the presence of the rs12248560 variant in a Chilean population and its accelerating effect on the pharmacokinetics of voriconazole in pediatric patients. From these data, it would be advisable to consider the variant of the patient prior to calculating the dosage of voriconazole.

## Introduction

Pediatric patients diagnosed with hemato-oncological diseases or who have had a solid organ transplant must undergo prolonged periods of chemotherapy and immunosuppressive therapy, which makes these patients susceptible to invasive fungal infections (IFIs)^1,2^. These infections are caused by the colonization of pathogens of the genus *Aspergillus* and *Candida* and are an important cause of morbimortality in pediatric patients. Overcoming these infections is crucial to the survival of these patients^3,4^.

Voriconazole is a broad-spectrum anti-fungal drug that belongs to the family of second-generation triazoles. It is used as a first-line treatment for IFIs, characterized by a high inter- and intra-patient pharmacokinetic variability. This condition makes its dosage difficult, which is why permanent therapeutic drug monitoring (TDM) is needed, focused mainly on achieving the correct dosage to obtain therapeutic plasma concentrations^5,6^.

Voriconazole is metabolized in the liver, mainly by two cytochrome P450 enzymes (CYP), CYP2C19 and CYP3A4, and flavin-containing monooxygenase 3 (FMO3)^7,8^ converting it to N-oxide voriconazole. This compound has minimal anti-fungal activity. The *CYP2C19* gene is highly polymorphic; to date more than 30 single nucleotide polymorphisms (SNPs) have been identified in this gene. However, few of these have been associated with any clinical response. The CYP2C19*17 allele is characterized by the presence of four SNPs (-3402 C > T, −806C > T, 99 C > T, 80161 A > G (I331V)). Also, CYP2C19*17 allele is also present in a low function allele CYP2C19*4B^9^. Several studies have shown that the presence of −806C > T SNP (rs12248560) is correlated with lower plasma concentrations of voriconazole^6,9–13^. The rs12248560 variant is associated with an ultra-rapid metabolizer phenotype of enzyme CYP2C19, which translates into a faster elimination of the drug in patients who receive treatment with standard doses of voriconazole with the consequent risk of therapeutic failure, making dosage corrections necessary^14–16^. Little is known of the effect of rs12248560 variant on pediatric patients; it is possible that genetic variants present in patient carriers could have a significant clinical impact on treatment with voriconazole, resulting in the spread of the infection and deterioration of the patient.

In Chile, there is no information about the frequency of rs12248560 variant and its impact on voriconazole pharmacokinetics. The aim of this work was to describe the frequency of rs12248560 variant in a Chilean population and to study its association with the pharmacokinetics of voriconazole in pediatric patients.

## Material and Methods

### Patients

**Figure 1:**
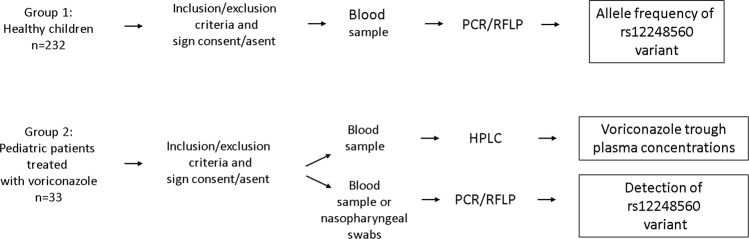
Methodological scheme of the study design.

In this work we analyzed two groups of children (Fig. 1). In order to determine the frequency of the rs12248560 variant in a Chilean population, a group of healthy children was enrolled (group 1). To evaluate the effect of the rs12248560 variant on voriconazole plasma concentrations, we included a group of immunocompromised children treated with voriconazole (group 2).

For group 1, 232 samples were collected from healthy Chilean children who attend the Dr. Luis Calvo Mackenna hospital (HLCM) for routine clinical testing. Children being treated with voriconazole were excluded. The ABO blood phenotype was obtained in 145 (63%) children in this group.

For group 2, 33 patients were included who received treatment with voriconazole, administered intravenously (IV) or orally (PO) between 2015 and 2017 years at the HLCM, Roberto del Río (HRR) and Base de Valdivia (HBV) hospitals. We excluded patients who presented pre-existing hepatic toxicity to treatment with voriconazole.

All patients and/or parents from groups 1 and 2 provided written informed consent to participate in the study approved by the Pediatric Scientific Ethics Committee of the Metropolitan East Health Service, the Ethics Committee of the Metropolitan North Health Service and the Valdivia Health Service. In addition, all methods were performed in accordance with Declaration of Helsinki.

### Nucleic acid extraction

Nucleic acids were extracted from blood samples using QIAamp® DNA blood Mini Kit (Qiagen) following the manufacturer’s instructions. For bone marrow transplant patients, the nucleic acids were extracted from nasopharyngeal swabs using the MagNa Pure Kit (Roche) according to the manufacturer’s instructions. Purified nucleic acids were stored at −20 °C for later use.

### Polymorphisms detection

PCR-RFLP was used to detect rs12248560 variant as described^3^. For the PCR amplification, the Go*Taq*® Flexi DNA polymerase enzyme from Promega was used. The restriction enzyme *LweI* was purchased from Thermo Fisher Scientific and used following the supplier’s specifications. The digestion fragments were visualized in 10% polyacrylamide gels. PCR-RFLP methods were validated by sequencing.

### Determination of the voriconazole plasma concentrations

For TDM, blood samples were taken in steady-state one hour before voriconazole administration according to the clinical protocols for antifungal treatment in each hospital. Voriconazole plasma concentration was determined by high performance liquid chromatography (HPLC) according to a previously validated method^17^ using an Agilent 1260 Infinity HPLC system with a VL diode array detector. Ketoconazole was used as the internal standard. Each run was done at 35 °C in the column with isocratic elution, a flow of 1 mL/min, 20 uL of sample injection and measured at a wavelength of 254 nanometers. Elution time for voriconazole and ketoconazole were 3.3 and 4.4 minutes, respectively. Recovery percentage of voriconazole was 101.6% ± 2% and both the repeatability (0.97%) and the reproducibility (1.73% and 1.77%) of the method were less than 2%.

### Statistical analysis

To determine the allelic frequency of the rs12248560 variant in a Chilean population (group 1), a sample size of 232 samples was calculated, assuming a frequency of this variant of 20% described for Caucasian population^18^, with 80% power, 5% error and a 95% confidence level. To compare the gender distribution between carriers and non-carriers of group 2, we used Fisher´s test. For the gender and ABO blood group comparison, as well as determination of the Hardy-Weinberg equilibrium, we used the chi-squared statistical test *(X*^*2*^). To determine the influence of the rs12248560 variant on voriconazole plasma concentrations, since one subject can have multiple plasma levels, a multilevel generalized linear model was created for correlated samples with gamma distribution and log link identity (distribution of the plasma concentration adjusted for dose and weight). A significant *p* value < 5% was considered. To determine the influence of the rs12248560 variant on the first voriconazole plasma level and to compare plasma concentrations measurements per patients, the Mann-Whitney statistical test was used. Statistical analysis was performed with Stata v.12.1 (StataCorp, 2011. Stata Statistical Software: Release 12. College Station, TX: StataCorp LP.).

## Results

### Allelic frequency of the rs12248560 variant in a Chilean population

Of the 232 children analyzed in group 1, 49.5% (115 patients) were girls, with an average of 5.5 ± 5.2 years of age. We found that 180 children (77.6%) were non-carriers of the rs12248560 variant, 49 children (21.1%) were heterozygous carriers for rs12248560 variant and only 3 children (1.3%) were homozygous carriers for rs12248560 variant (Table 1). With these genotypic frequencies, an allelic frequency of 12% for the rs12248560 variant was obtained. In addition, it was established that this population is in the Hardy-Weinberg equilibrium.

**Table 1:** Demographic and pharmacokinetic data of patients with voriconazole treatment.

	Non-Carriers	Carriers	*p* Value
Total patients	22	11	
Female	10	7	0.465
Male	12	4	
Age median (years) [IQR]	7.5 [2.8–10.5]	9 [7,8–10,5]	0.135
Weight median (kg) [IQR]	23.5 [14,1–28,8]	27.7 [25,0–32,0]	0.0217
Plasma concentrations measurement/patients median [IQR]	1.5 [1–2,75] (*n* = 22)	1.0 [1–2] (*n* = 11)	0.615
Dose median (mg/kg/day) [IQR]	14.9 [10,0–16,0] (*n* = 81)	14.6 [9,3–18,0] (*n* = 49)	0.9
All the dose-corrected plasma concentrations (µg/mL/mg/kg/day) mean [SD]	0.046 [0,174] (*n* = 81)	0.034 [0,07] (*n* = 49)	0.011
First voriconazole plasma concentration median (µg/mL) [IQR]	1.12 [0,39–3,92] (*n* = 22)	0.37 [0,16–1,19] (*n* = 11)	0.054
First dose-corrected voriconazole concentration median (µg/mL/mg/kg/day) [IQR]	0.10 [0,02–0,34] (*n* = 22)	0.029 [0,02–0,08] (*n* = 11)	0.059
Diagnosis			
ALL	13	6	
AML	5	5	
Osteosarcoma	1	—	
Liver transplant	1	—	
Testicle tumor	1	—	
SCID	1	—	

IQR, interquartile range; SD, Standard deviation; ALL, acute lymphocytic leukemia; AML, acute myeloid leukemia; SCID, Severe Combined Immunodeficiency.

No statistical differences were found in the ABO blood group between the children included in this study and a Chilean population, indicating that the study population was representative (Supplementary Fig. 1).

### Allelic frequency of the rs12248560 variant in patients treated with voriconazole

The presence of the rs12248560 variant was studied in 33 patients treated with voriconazole (group 2). The demographic and pharmacokinetic data are shown in Table 1. The rs12248560 variant was found that 66.7% (22 patients) of the patients were non-carriers of rs12248560 variant, 27.3% (9 patients) were heterozygous carriers of rs12248560 variant and 6% (2 patients) were homozygous carriers of rs12248560 variant.

### Carrier of rs12248560 variant presented lower voriconazole plasma concentrations than non-carriers

**Figure 2:**
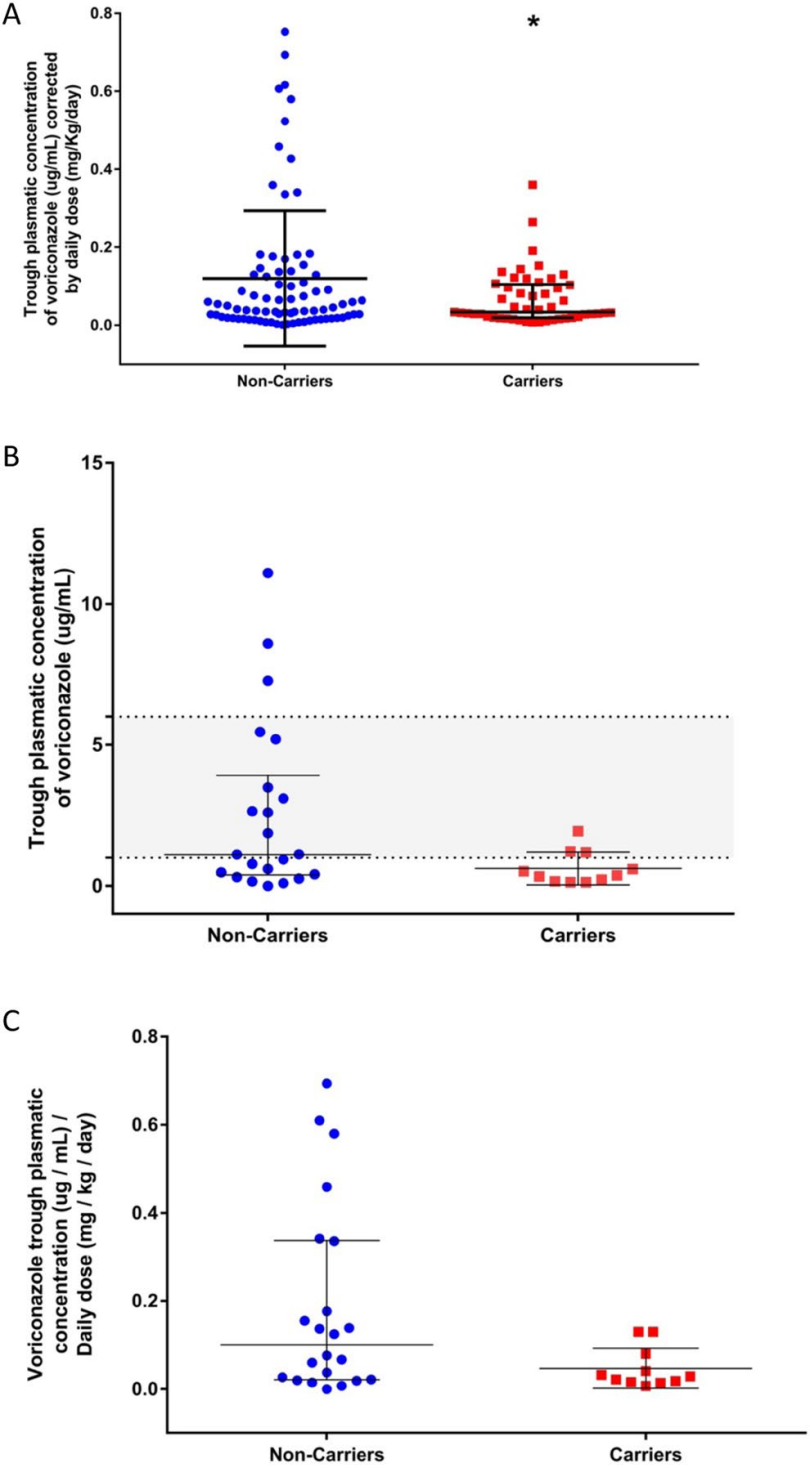
Voriconazole plasma concentration in carriers or non-carriers of the rs12248560 variant. (**A**) Dose-corrected voriconazole plasma concentrations of the 33 patients are shown. The carriers had a mean of 0.034 µg/mL/mg/kg/day and showed significantly lower concentrations than non-carriers (*p* = 0,011), whose mean was 0.046 µg/mL/mg/kg/day. The mean and the standard deviation for each genotype are plotted. (**B**) First trough plasma concentrations of voriconazole, without dose correction, reached by the patients studied (*p* = 0.054). Almost all the concentrations belonging to the carriers of the rs12248560 variant were sub-therapeutic (median 0.37 µg/mL), whereas for the non-carriers the concentrations were spread out (median 1.12 µg/ml). The medians and interquartile ranges are plotted and the therapeutic range (between 1 and 6 µg/mL) is highlighted. (**C**) First dose-corrected voriconazole concentration of the 33 patients is shown. The median for each genotype were 0.10 and 0.029 µg/mL/mg/kg/day for non-carriers and carriers of the rs12248560 variant, respectively (*p* = 0.059).

We compared 81 dose-corrected voriconazole plasma concentrations of 22 non-carriers of rs12248560 variant and 49 plasma concentrations from 11 carriers of the rs12248560 variant. It is important to note, that patients included in our study were grouped by presence or absence of the rs12248560 variant (carriers and non-carriers) and not based on CYP2C19 phenotype, since only two patients were homozygous for the rs12248560 variant with one level each. We found that carriers of the rs12248560 variant presented significantly lower voriconazole plasma concentrations than non-carriers (*p* = 0,011; Fig. 2A).

The voriconazole plasma concentrations without dose correction were studied (first plasma level). It was observed that almost all the carriers of the rs12248560 variant presented the first sub-therapeutic trough plasma concentration with a median of 0.37 µg/mL, whereas in the non-carriers there was a wide spread in the concentrations with a median of 1.12 µg/mL (Fig. 2B).

Finally, the first trough voriconazole dose-corrected plasma concentrations for each genotype were compared. The carriers of the rs12248560 variant had a median 0.029 µg/mL/mg/kg/day, whereas the non-carriers had 0.10 µg/mL/mg/kg/day (*p* = 0.059; Fig. 2C).

## Discussion

In this study we examined the association of the rs12248560 variant on voriconazole plasma concentrations in immunocompromised Chilean pediatric patients. In addition, the allelic frequency of this variant in a Chilean population was obtained. The rs12248560 variant has been studied in other populations^19^. In a population of 285 subjects in the north of Spain, 14.9% of them were carriers of the rs12248560 variant, a value similar to the 12% obtained in our study^20^. Similarly, data obtained from the 1000 Genomes Project showed a frequency 12% of the rs12248560 variant in the American population. In another study in the north (Denmark, Sweden, Norway), east (Poland) and south (France) of Europe, the allelic frequencies of this variant was between 15 and 27%. This frequency was similar to what has been described in Africa (Ethiopian, Nigerian, Afro-American), a frequency reported as being between 18 and 20%, whereas for Asian groups (Korean, Chinese and Japanese), frequencies between 2 and 4% were described^21^.

We found differences in voriconazole plasma concentrations in carriers of the rs12248560 variant compared to non-carriers. Also, analyzing the first trough voriconazole plasma concentrations revealed that plasma concentrations found in rs12248560 variant carriers were lower than the non-carriers. The same association has been reported in immunosuppressed hemato-oncology pediatric patients^6,12^, demonstrating that the heterozygote patients have lower plasma concentrations than non-carriers. In another study performed on 72 adult patients with aspergillosis^22^, it was determined that rs12248560 variant carriers presented statistically lower voriconazole plasma concentrations than non-carriers. The pharmacokinetics of voriconazole in 20 healthy adults after a single oral dose of 200 mg showed that rs12248560 variant carriers had a maximum concentration, AUC and clearance of voriconazole significantly lower than non-carriers^10^.

Early determination of the allelic rs12248560 variant and its consideration in guiding the voriconazole dosage in patients made it possible to reduce the time needed to reach therapeutic concentrations from 29 to 6.5 days in carriers of the rs12248560 variant and from 25 to 9 days in homozygous patients for the wild-type variant CYP2C19*1. In addition, there was a reduction in patients with an elevation of liver enzymes and suspension of the treatment due to toxicity^12^. A recent report established that the CYP2C19 enzyme genotype is the most important interpersonal variability factor that affects the plasma concentrations of voriconazole^23^. In this regard, clinical guidelines for voriconazole therapy based on the *CYP2C19* genotype have recently been published providing evidence for the use and dosage of this drug^19^. Implementation of genotype-targeted therapy together with adequate therapeutic monitoring in accordance with clinical recommendations could produce even shorter dwell times or reduce the hospital stay, avoid transfer to high complexity units and prolonged drug treatment with voriconazole, lower the request for imaging or laboratory examinations, and so forth.

Our study has limitations. An interindividual variability in voriconazole plasma levels has been described. Age, gender, drug route and some co-morbidities have been shown to affect the voriconazole plasma concentration^19,24,25^. In our study no differences were found in age and gender between carriers and non-carriers of the rs12248560 variant in patients treated with voriconazole (Table 1). Drug route and co-morbidities were not analyzed. Another limitation is that drug interactions, efficacy and toxicity of voriconazole and its association with clinical outcome were not analyzed in our study. Is important to note, in this study we did not analyze the involvement of others *CYP2C19* variants or others enzymes (CYP3A4 and FMO3) that affects the plasma concentrations of voriconazole that might explain similar plasma concentrations between carriers and non-carriers of rs12248560 variant. The main goal of our study was to find evidence of the impact of the rs12248560 variant on the voriconazole plasma concentration. Future prospective age-matched studies including the variables mentioned above are needed to validate our findings in order to consider CYP2C19*17 genotyping for voriconazole therapeutic monitoring.

In conclusion, we report the frequency of the rs12248560 variant in a Chilean pediatric population and the involvement of this variant in the voriconazole metabolism of immunocompromised patients. Our data might help in the implementation of CYP2C19 genotyping to individualize starting doses of voriconazole.

## Supplementary information

Supplementary information
