# Supplementary material for: Frequency of the CYP2C19*17 polymorphism in a Chilean population and its effect on voriconazole plasma concentration in immunocompromised children
Source: Sci Rep. 2019 Jun 20;9:8863. doi: 10.1038/s41598-019-45345-2 (PMC6586657; doi:10.1038/s41598-019-45345-2)

Frequency of the *CYP2C19*17* polymorphism in a Chilean population and its effect on voriconazole plasma concentration in immunocompromised children

Espinoza N.[1], Galdames J.[1], Navea D.[1], Farfán MJ[1,2*] and Salas C.[1*]

[1] Laboratorio Clínico, Hospital Dr. Luis Calvo Mackenna
[2] Departamento de Pediatría y Cirugía Infantil, Campus Oriente, Hospital Dr. Luis Calvo Mackenna, Facultad de Medicina, Universidad de Chile

Supplementary Figure 1. The frequencies of the ABO blood phenotype described for the Chilean population are shown in (A), and those obtained in the sample of Chilean children (B). The blood phenotype was determined in 65% (151/232) of the analyzed samples from Chilean children; no statistically significant differences were found (X^2 = 2.135, p value = 0.5449, with 3 degrees of freedom).

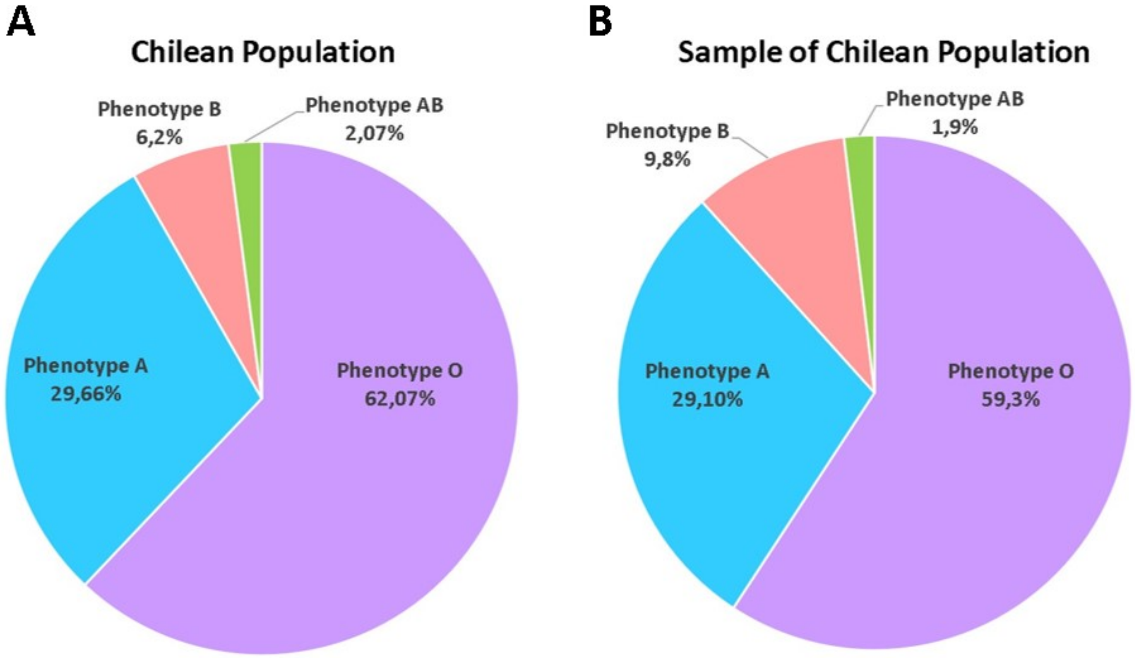

Supplement: Supplementary file 1 — Supplementary information [file 41598_2019_45345_MOESM1_ESM.pdf]
